# Adherence to a snacking dietary pattern is decreasing in Colombia among the youngest and the wealthiest: results of two representative national surveys

**DOI:** 10.1186/s12889-019-8057-6

**Published:** 2019-12-19

**Authors:** Oscar F. Herrán, Eduardo Villamor, Doris C. Quintero-Lesmes

**Affiliations:** 1 Facultad de Salud (UIS), Escuela de Nutrición y Dietética, Universidad Industrial de Santander, Carrera 32 No. 29-31, Santander Bucaramanga, 680002 Colombia; 2 Department of Epidemiology, University of Michigan School of Public Health, Ann Arbor, MI USA; 3 Oficina Diseño y Desarrollo, Fundación Cardiovascular de Colombia, Floridablanca, Colombia

**Keywords:** Food pattern, Snack pattern, Intake of snack, Children, Adults, Colombia

## Abstract

**Background:**

A common recommendation is to reduce the consumption of snack food and replace this consumption with nutrient-dense foods. The objective was to assess whether in Colombian children and adults there were changes in the consumption of the snack dietary pattern (SP) in the 5 years 2010–2015. In addition, this study aimed to establish the relationship between the SP and some biological, socioeconomic, and geographic variables in Colombia, South America.

**Methods:**

Based on a Food Frequency Questionnaire (FFQ) applied both in 2010 and 2015 in the national nutritional situation surveys, the adherence to the snack consumption pattern was established through factor analysis. The change in the adherence of consumption to the SP was established for the five-year period [2015 minus 2010], using multiple linear regression models. Crude and adjusted differences were estimated by the following covariables: sex, age, marital status, food security, wealth index, ethnicity, education of the head of the household, area and region. In total, 37,981 subjects were analyzed. In 2010, 10,150 children (5 to 17 years old) and 5145 adults (18 to 64 years old) were included, and in 2015, 13,243 children and 9443 adults.

**Results:**

In children, the adjusted difference in the adherence to SP was − 0.37 (95% CI: − 0.42, − 0.32). In adults, the adjusted difference in the adherence to SP was − 0.27 (95% CI: − 0.31, − 0.24). In all categories of covariables, consumption decreased, for all *p* < 0.0001. In children, the decrease in consumption was inversely associated with height-age. The decrease was smaller at the extremes of the BMI distribution, Z < -2 and Z > 2. The decrease in consumption was directly associated with the level of food security in the home and the wealth index. In adults, the decrease in consumption was inversely related to age and was directly related to the level of food security of the household, wealth index, and education level. The BMI decrease was greater in subjects with 18.5–24.9. In subjects with 30+, it was lower than in subjects with 25.0–29.9.

**Conclusions:**

In the 5 years 2010–2015, snack consumption is decreasing. The region, the richest subjects, those with adequate BMI, and in households with more educated heads of household, achieved a greater decrease in SP.

## Background

Nutritional epidemiology has studied the relationship between diet and disease from the perspective of nutrients, food, and eating patterns [1–9]. Autochthonous diets recognized for their cardioprotective effects, such as the Mediterranean [10, 11] or Eskimo or Inuit [12] diets, are the expression of what we now understand as traditional or ancestral food patterns. Evidence suggests that adhering to traditional dietary patterns - regardless of the geographical context - protects or delays the undesirable effects of ultraprocessed foods and other patterns on health, such as the so-called “western” diet, which consists of a high content of refined foods, sugar, saturated fat, ultraprocessed ingredients and, in general, items considered snack foods [13–16].

In countries with high and medium incomes, snack consumption contributes to between 20 and 40% of the total energy/day [17–19]. Snack consumption in children and adults has been associated with the development of adiposity, annual changes in body mass index (BMI), and subcutaneous fat (SCF) [20–22]; it is also positively associated with waist circumference and SCF thickness in overweight and obese men and women [23, 24]. The contradictory evidence of these associations is the result of studies with cross-sectional data, where the overweight subjects report less snack consumption and the different ways in which the consumption of snacks is defined and measured [21, 25, 26]. Despite the obvious limitations in defining and measuring snack consumption [25], a common recommendation is to reduce the consumption of snack food and replace this consumption with nutrient-dense foods.

In Colombia, based on the National Nutrition Surveys (ENSIN, 2010 and 2015) [27, 28], it was recently established that three patterns of food consumption coexist; traditional/starch, fiber/dairy and snack. The objective of this study was to establish whether there were changes in adherence to the consumption of the snack pattern (SP) in Colombian children and adults in the 2010–2015 quinquennium. In addition, this study aimed to establish the relationship between the SP and some biological, socioeconomic, and geographic variables.

## Methods

### Population studied

During the last 8 years in Colombia, the Colombian Family Welfare Institute (Instituto Colombiano de Bienestar Familiar, ICBF) has performed two national surveys of the nutritional situation (ENSIN, 2010 and 2015) [27, 28]. The details of these ENSINs have already been published. In summary, the ENSINs are designed to select a representative sample for the target population using a stratified multistage sampling technique. All 33 geodemographic units are grouped based on similar geographic and sociodemographic characteristics. The municipalities were randomly selected and the representation of them in the sample is proportional to the size of their population. The stratum corresponds to a set of municipalities. Clusters of approximately 10 households are randomly selected within these strata, and members of households are invited to participate. In 2010, the survey included 50,670 households, representing 4987 clusters of 258 strata. In 2015, the survey included 44,202 households, representing 4739 clusters of 177 strata. Consent to participate was obtained by the ICBF before the recruitment of the households and subjects.

### Data sources

In both surveys, trained personnel administered questionnaires to the head of the household to obtain sociodemographic information on food security and the level of household wealth. In addition, nutritionists applied in a randomly selected sub-sample a Food Frequency Questionnaire (FFQ). Children under 12 years of age were assisted by their caregivers to respond to the FFQ. The checklist of food and food groups was designed by nutritionists based on the nutrition problems identified in the ENSIN-2005. The response section was adapted from two reproducibility and validity studies of FFQs used in the Colombian population [29, 30]. The facial validity of all the items on the checklist was guaranteed. The anthropometric measurements were also taken by trained nutritionists using standardized techniques and calibrated equipment. Height was obtained with stadiometers (Shorr Productions LCC, Olney, MD, USA) and was measured to the nearest millimeter. Weight was obtained with SECA scales (model 872 in 2010 and model 874 in 2015) and was measured to the nearest 100 g.

In 2010, the FFQ was applied to 7138 subjects between 18 and 64 years old; in 2015, it was applied to 11,530 subjects. Of these subjects, we excluded pregnant women (in 2010, *n* = 1679; in 2015, *n* = 1134), those who practiced prescribed diets (in 2010, *n* = 255; in 2015, *n* = 304), and with BMI [kg/m^2^] less than 14 or greater than 60 for considering them extreme and not plausible. In addition, in 2010, the FFQ was applied to 10,756 subjects between 5 and 17 years old, and in 2015, it was applied to 10,092 subjects. Of these, we excluded pregnant girls (in 2010, *n* = 257; in 2015, *n* = 159), those who practiced prescribed diets (in 2010, *n* = 128; in 2015, *n* = 126). Also, children’s BMI data outside the range of − 6 to 6 z scores based on the WHO chart were excluded from this study [31]. In total, 37,981 records were analyzed. In 2010, 10,150 children (5 to 17 years old) and 5145 adults (18 to 64 years old) were included, and in 2015, 13,243 children and 9443 adults were included.

The FFQ administered in the ENSIN had 28 food items, all of which were included in the analyses of dietary patterns. In 2010, the 10 responses in the FFQ regarding the frequency of consumption of 28 food items were converted to a continuous variable: “times/day” (The details of this procedure can be requested from the authors). This variable was assessed using a factor analysis, and three consumption patterns were established. The three established patterns were the SP [package foods, sweets, soft drinks (powder, box, bottle), fast food, butter, sausages, chicken starters], fruit-vegetable/dairy [milk, cheese, kumis, yogurt, cream cheese, raw vegetables, cooked vegetables, whole fruits, fruits in juice, bread, arepa or cookies, whole-grain, chicken, black pudding or beef viscera, low calorie foods (light), tuna or sardines], and traditional/starch [panela, sugar, honey, rice or pasta, fried foods, dry beans, tubers or banana, eggs, beef, veal, pork, fish or seafood, coffee or tea]. In 2015, similar to 2010, the responses of the frequency of consumption in the FFQ became a continuous variable measured in “times/day”, and the same 28 foods of the FFQ were analyzed factorially to keep the results between the surveys comparable.

Factor analysis is a data reduction technique, based on the correlations between them. Here, 28 items were grouped into 3 new variables called food patterns. This factorial solution is itself a mathematical model. Nutritional epidemiology very often uses this analysis technique to better represent the consumption of the subjects. To make the values ​​in the new variables comparable, these are expressed as Z scores. Thus, a value of 0 means that a subject has the average value in the consumption of the other subjects. Z scores allow classifying subjects among them based on your consumption. The value of Z (Z Score) here is called “adherence” to the consumption pattern. Based on the above, it can be affirmed that the dietary intake of a subject in Colombia has three patterns, which complement each other. The details of the procedure to establish food patterns based on ENSIN-2010 have already been published [26]. The standardized scores obtained based on the frequency of consumption and the factorial loads were declared as the adherence that each subject had to each of the three established patterns.

The variable of interest was adherence in consumption to the SP. The primary exposure was the year of conducting the survey (2010 or 2015). In addition, for each survey, other covariables were considered in relation to adherence to the SP, including sex, age, height, BMI (in children, according to WHO) [31], marital status, state of food security in the home, wealth index, ethnicity, education of the head of the household, the level of urbanism, and the geographical region where the subjects live. The level of urbanism was categorized as those who live in urban areas of large cities and those who live in rural areas. The rural category included suburban population centers close to small cities, principal rural areas distant from small cities and dispersed or very distant populations of rural principal settlements. The food security status of the home was established using the Latin American and Caribbean Scale of Food Security (ELCSA), a modified version of the Community Childhood Hunger Identification Project, which has undergone successive adaptation and validation processes in Colombia [32]. Wealth was established using the index designed for the international population and health survey [33]. This index was constructed in the same way for the two surveys through an analysis of the main components with household information, which included, among others, the type of construction material of the dwelling, the characteristic of the sanitary services, and in general, the goods and services that the household has. The first component was used to create the index as a variable of continuous type (Z score), which is assigned to each subject within the household. The highest values represent the wealthiest subjects. The wealth index was categorized by incorporating the complex design of the sample into quintiles according to the distribution reached among all the participants in each survey.

### Statistical analysis

All analyses were conducted using the analysis routines for complex sample designs of Stata software, version 14.1 [34]. An analysis was conducted to estimate the average adherence to SP in the covariable categories. In addition, using a multiple linear regression with adherence to SP as the dependent variable, we estimated the adjusted differences for each of the categories in all covariates and their 95% confidence intervals (95% CIs). To obtain the adjusted differences, a new term was created as the cross product between the year and each category of the covariates (interaction). The adjusted differences incorporated the complex design of the sample, and the multiple regression model included the following covariates: sex, age, marital status, food security, wealth index, ethnicity, education of the head of household, area, and region. Finally, using a multiple linear regression, crude and adjusted differences and their respective 95% CIs were estimated between the frequency/day of consumption for the main items that compose the SP.

### Institutional review board

The authors declare that all procedures that contributed to this work comply with the ethical standards of the Declaration of Helsinki, revised in 2008. The research ethics committee of the National Institute of Health of Colombia approved the survey protocol, and all participants provided informed consent. The health research ethics committee of the Universidad Industrial de Santander states that anonymized data analyses are exempt from review.

## Results

### In children

In 2010, 49.3% of the subjects were girls. The mean age ± SE of the subjects was 11.8 ± 0.05 years, which differed by sex (*p* = 0.041). In 2015, 49.0% of the subjects were girls. The average age of the subjects was 11.0 ± 0.07 years, which differed by sex, with girls being an average of 3 months older (*p* = 0.001).

**Fig. 1:**
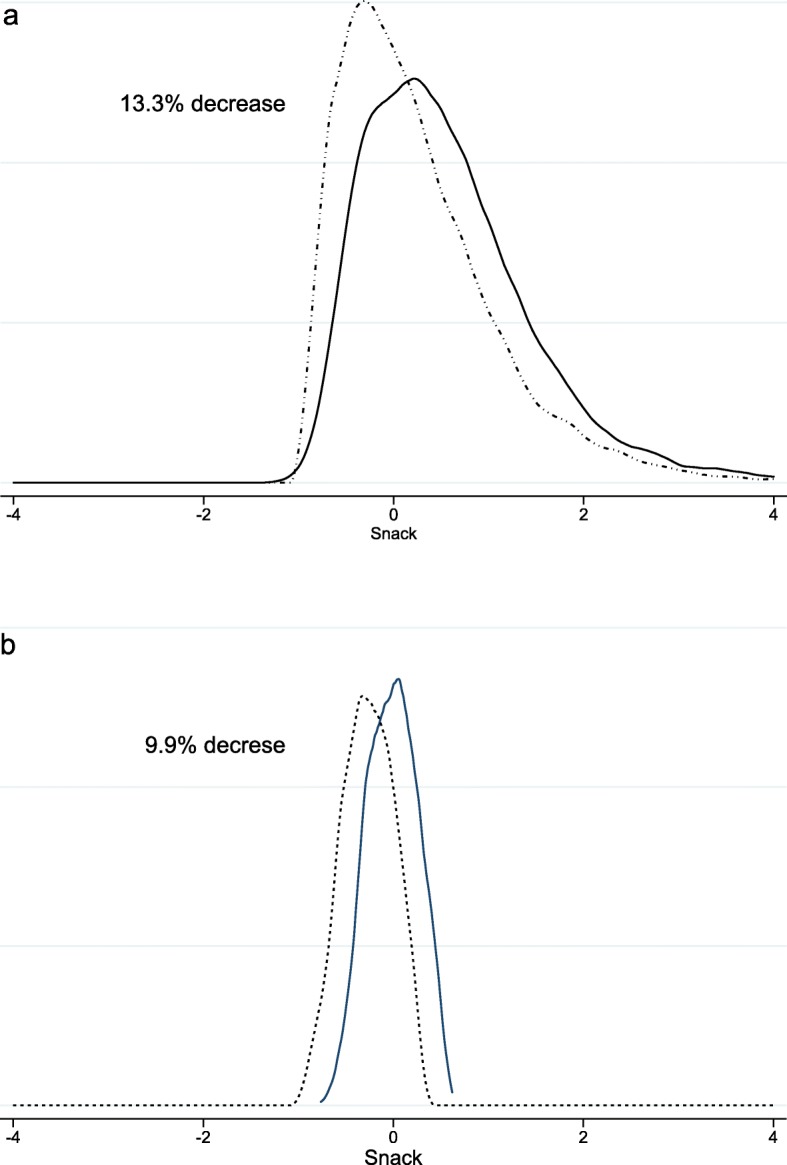
Adherence to Snack food consumption pattern (Z) in Children 5 to 17*y* (**a**) and Colombian adults 18-64*y* (**b**); in 2010 ____ in 2015…….. Figure 1, graphically presents the concept of food transition in the consumption of snack pattern, both in children and adults. This phenomenon difficult to graph, is only possible when repeated measurements are made over time guaranteeing the same methodology. The reduction of consumption plotted in normal curves, allows the estimation of the reduction through the calculation of areas under the curves

The adjusted difference in adherence to the SP was − 0.37 (95% CI: − 0.42, − 0.32, *p* < 0.0001). In each year, age, height/age, and wealth index were directly associated with consumption. Those who live in urban areas consumed more. In all categories of variables, consumption decreased (*p* < 0.0001 for all). In this period, the decrease was greater in boys and in those who live in urban areas. The decrease in consumption was inversely associated with height-age. The decrease was smaller at the extremes of the BMI distribution, Z < -2 and Z > 2. The decrease in consumption was directly associated with the level of food security in the home and the wealth index (Table 1). The decrease in SP consumption in children was 13.3% (Fig. 1a).

**Table 1:** Differences adjusted to the score of adherence to the Snack food consumption pattern (Z score). Colombian children (5 to 17 y) in 2010 and 2015

Variable	2010				2015				Adjusted difference^b^ 2015–2010	*P* Interaction
	n^a^	Mean	SE	*P* value	n^a^	Mean	SE	*P* value		
Sex				0.375				0.014		< 0.0001
Male	5154	0.66	0.02		6753	0.29	0.04		−0.40 (− 0.46, − 0.34)	
Female	4996	0.68	0.02		6490	0.35	0.04		− 0.34 (− 0.40, − 0.28)	
Age group (y)				< 0.0001				< 0.0001		< 0.0001
Children (5–10)	3794	0.53	0.02		4627	0.21	0.04		−0.36 (− 0.43, − 0.30)	
Teenagers (11–17)	6356	0.75	0.02		8616	0.41	0.04		−0.37 (− 0.44, − 0.31)	
Age (y)				< 0.0001				< 0.0001		< 0.0001
5–8	2481	0.51	0.02		3274	0.21	0.05		−0.35 (− 0.42, − 0.28)	
9–11	1949	0.57	0.03		2090	0.25	0.04		− 0.36 (− 0.44, − 0.28)	
12–15	3480	0.78	0.02		5064	0.44	0.04		−0.38 (− 0.46, − 0.31)	
16–17	1880	0.74	0.03		2815	0.40	0.04		−0.36 (− 0.45, − 0.28)	
Stunting (Height/Age)				< 0.0001				< 0.0001		< 0.0001
No	8820	0.69	0.01		12,010	0.34	0.04		−0.37 (− 0.42, − 030)	
Yes (Z < -2)	1324	0.51	0.04		1227	0.08	0.05		−0.42 (− 0.54, − 0.31)	
Nutritional status (BMI)^c^				0.068				0.728		< 0.0001
No	4190	0.79	0.02		11,075	0.33	0.04		−0.40 (− 0.47, − 0.33)	
Overweight (≥25)	736	0.74	0.05		1691	0.27	0.03		−0.43 (− 0.54, − 031)	
Obesity (≥30)	201	0.65	0.08		477	0.37	0.09		−0.31 (− 0.51, − 0.10)	
Household food insecurity				< 0.0001				0.186		< 0.0001
No	3004	0.82	0.02		4108	0.37	0.04		−0.46 (− 0.53, − 038)	
Light	3768	0.63	0.02		4686	0.30	0.04		−0.36 (− 0.43, − 0.30)	
Moderate	1973	0.56	0.03		2512	0.37	0.04		−0.31 (− 0.39, − 0.22)	
Severe	1393	0.52	0.04		1937	0.31	0.08		− 0.26 (−0.40, − 012)	
Wealth index quintile				< 0.0001				< 0.0001		< 0.0001
1- poorest	3595	0.33	0.02		2839	−0.05	0.04		−0.33 (− 0.41, − 025)	
2	2462	0.58	0.03		2794	0.25	0.05		−0.30 (− 0.39, − 0.20)	
3	1815	0.82	0.03		2762	0.36	0.03		−0.38 (− 0.46, − 0.29)	
4	1303	0.82	0.03		2525	0.45	0.04		−0.35 (− 0.43, − 0.26)	
5- wealthiest	975	0.93	0.04		2323	0.44	0.05		−0.48 (− 0.59, − 0.37)	
Ethnicity				< 0.0001				0.170		0.003
Mestizo	7702	0.68	0.02		10,620	0.32	0.04		−0.38 (− 0.43. -0.33)	
Black/Afro	1103	0.74	0.04		1393	−0.14	0.06		−0.71 (− 0.82, − 0.59)	
Indigenous	1234	0.16	0.05		1125	0.55	0.06		0.20 (0.05, 0.35)	
Area				< 0.0001				< 0.0001		< 0.0001
Urban	6549	0.82	0.02		9723	0.44	0.04		−0.39 (− 0.45, − 032)	
Rural^d^	3601	0.28	0.02		3520	−0.01	0.03		−0.30 (− 0.37, − 0.22)	
Region				0.499				0.287		< 0.0001
Central	2335	0.70	0.03		3189	0.40	0.11		−0.35 (− 0.52, − 0.19)	
Atlantic	2292	0.76	0.03		2410	0.36	0.05		−0.45 (− 0.54, − 0.36)	
Oriental	1481	0.57	0.03		2272	0.23	0.04		−0.42 (− 0.50, − 0.33)	
Pacific	1406	0.43	0.03		1659	0.27	0.11		−0.18 (− 0.34, − 0.02)	
Bogotá	524	0.94	0.04		877	0.37	−0.00		− 0.58 (− 0.66, − 0.49)	
National territories	2112	0.38	0.03		2836	0.02	0.04		−0.42 (− 0.51, − 0.33)	

^a^In 2010 *n* may be less than 10,150 for missing values. In 2015 *n* may be less than 13,243 for missing values

^b^Adjusted difference and 95% confidence interval achieved in a linear regression model with the score of adherence (Z score) to the snack pattern as a dependent variable and predictors that include indicator variables for each sociodemographic correlates, year 2015 (2010 as reference) and cross-product (interaction) terms between year and indicator variables of the correlate. In addition, the linear regression model was adjusted by the following covariables; sex, age, food security, wealth index, ethnicity, area and region. The complex sampling survey design was taken into account in all multivariate regression models

^c^Based on equivalent cut-off points using the IOFT classification

^d^The rural category included suburban population centers close to small cities, towns in rural areas distant from small towns, and disperses or very distant from rural towns

### In adults

In 2010, 55.2% of the subjects were women. The mean age ± SE of the subjects was 37.5 ± 0.24 years, which did not differ by sex. In 2015, 55.1% of the subjects were women. The mean age of the subjects was 37.8 ± 0.23 years, which differed by sex, with women being an average of 2 years older (*p* < 0.0001).

The adjusted difference in adherence to SP was − 0.27 (95% CI: − 0.31, − 0.24, *p* < 0.0001). In each year, age was inversely associated with consumption and was directly associated with the level of food security of the household, wealth index, and education level. The BMI decrease was greater in subjects with 18.5–24.9, in subjects with 30+, it was lower than in subjects with 25.0–29.9. Men, married people, and those who live in urban areas consumed more. In all categories of variables, consumption decreased (*p* < 0.0001 for all). In the period, the decrease was greater in men, single people, and those who live in urban areas (Table 2). The decrease in SP consumption in adults was 9.9% (Fig. 1b).

**Table 2:** Differences adjusted to the score of adherence to the Snack food consumption pattern (Z score). Colombian adults (18-64y) in 2010 and 2015

Variable	2010				2015				Adjusted difference^b^ 2015–2010	*P* Interaction
	n^a^	Mean	SE	*P* value	n^a^	Mean	SE	*P* value		
Sex				< 0.0001				< 0.0001		< 0.0001
Male	2303	0.12	0.03		4236	−0.16	0.03		− 0.32 (− 0.38, − 0.26)	
Female	2842	− 0.06	0.02		5207	− 0.28	0.02		−0.24 (− 0.28, − 0.19)	
Age (years)				< 0.0001				< 0.0001		< 0.0001
18–24	1124	0.62	0.05		1806	0.14	0.04		−0.52 (− 0.63, − 0.41)	
25–34	1283	0.17	0.03		2262	− 0.12	0.03		−0.31 (− 0.38, − 0.23)	
35–44	1150	− 0.15	0.02		1964	− 0.31	0.02		− 0.18 (− 0.25, − 011)	
45–54	962	− 0.34	0.02		1990	−0.42	0.02		−0.11 (− 0.16, − 0.05)	
55–64	626	−0.44	0.02		1421	−0.56	0.02		−0.13 (− 0.18, − 0.07)	
Talla (cm)				< 0.0001				< 0.0001		< 0.0001
< 150	606	−0.30	0.04		1031	−0.41	0.03		−0.09 (− 0.17, − 0.01)	
150–159.9	1953	− 0.08	0.02		3478	−0.28	0.02		−0.23 (− 0.28, − 0.18)	
160–169.9	1711	0.06	0.03		3271	−0.20	0.02		−0.30 (− 0.36, − 0.24)	
170 +	848	0.35	0.05		1591	−0.07	0.03		−0.43 (− 0.53, − 0.33)	
Body mass index				< 0.0001				< 0.0001		< 0.0001
< 18	76	0.17	0.13		136	0.27	0.23		–	
18–24.9	2419	0.14	0.03		3965	−0.16	0.02		−0.33 (−0.39, − 0.27)	
25–29.9	1842	−0.09	0.02		3474	−0.29	0.02		−0.24 (− 0.29, − 0.19)	
30 +	808	−0.13	0.04		1868	−0.29	0.03		−0.21 (− 0.28, − 0.13)	
Marital status				< 0.0001				< 0.0001		< 0.0001
Married/Living together	3140	−0.12	0.02		5663	−0.30	0.02		−0.22 (− 0.26, − 0.18)	
Unmarried/single	2040	0.21	0.03		3780	−0.12	0.02		−0.35 (− 0.41, − 0.29)	
Food security				0.003				0.124		< 0.0001
Food secure	1947	0.08	0.03		3559	−0.23	0.02		−0.31 (− 0.37, − 026)	
Mild food insecurity	1937	−0.01	0.03		3389	−0.25	0.02		−0.28 (− 0.34, − 0.23)	
Moderate food insecurity	754	−0.07	0.04		1544	−0.18	0.03		−0.17 (− 0.25, − 0.09)	
Severe food insecurity	505	−0.05	0.07		949	−0.17	0.04		−0.17 (− 0.32, − 0.02)	
Wealth index quintile				< 0.0001				< 0.0001		< 0.0001
1- poorest	1399	−0.23	0.03		1575	−0.44	0.02		−0.19 (− 0.26, − 0.13)	
2	1196	−0.04	0.04		1774	−0.33	0.03		−0.26 (− 0.35, − 0.18)	
3	1053	0.08	0.04		1793	−0.16	0.03		−0.24 (− 0.31, − 0.16)	
4	819	0.06	0.03		1969	−0.17	0.02		−0.25 (− 0.32, − 0.17)	
5- wealthiest	678	0.16	0.04		2332	−0.20	0.02		−0.36 (− 0.44, − 0.28)	
Ethnicity				0.151				0.066		< 0.0001
Mestizo	4033	0.02	0.02		8054	−0.23	0.02		−0.26 (− 0.30, − 0.23)	
Black/Afro	557	0.12	0.05		559	−0.42	0.06		−0.46 (− 0.59, − 0.32)	
Indigenous	486	−0.23	0.04		809	−0.14	0.03		−0.02 (− 0.11, 0.07)	
Education of the head				< 0.0001				0.888		< 0.0001
Preschool or less	360	−0.27	0.04		42	−0.01	0.02		−0.13 (− 0.36, 0.10)	
Primary	2216	−0.13	0.02		6223	−0.23	0.02		−0.15 (− 0.20, − 0.11)	
Secondary	1708	0.14	0.03		2551	−0.16	0.02		−0.33 (− 0.40, − 0.27)	
Post-secondary	680	0.17	0.04		566	−0.48	0.02		−0.44 (− 0.54, − 0.33)	
Area				< 0.0001				< 0.0001		< 0.0001
Urban	3643	0.10	0.02		7113	−0.17	0.02		−0.28 (− 0.32, − 0.24)	
Rural^c^	1502	− 0.26	0.02		2330	−0.42	0.02		−0.18 (− 0.24, − 0.12)	
Region				0.228				0.076		< 0.0001
Central	1363	0.06	0.03		2298	−0.24	0.04		−0.29 (− 0.38, − 0.20)	
Atlantic	1133	0.10	0.03		2136	−0.16	0.04		−0.31 (− 0.38, − 0.23)	
Oriental	681	−0.06	0.05		1726	−0.19	0.03		−0.16 (− 0.27, − 0.05)	
Pacific	809	−0.16	0.03		1203	−0.29	0.05		−0.14 (− 0.25, − 0.04)	
Bogotá	328	0.14	0.05		663	−0.24	0.00		−0.40 (− 0.49, − 0.30)	
National territories	831	−0.14	0.03		1417	−0.39	0.03		−0.32 (− 0.42, − 0.22)	

^a^In 2010 *n* may be less than 5145 for missing values. In 2015 *n* may be less than 9443 for missing values.

^b^Adjusted difference and 95% confidence interval achieved in a linear regression model with the score of adherence (Z score) to the snack pattern as a dependent variable and predictors that include indicator variables for each sociodemographic correlates, year 2015 (2010 as reference) and cross-product (interaction) terms between year and indicator variables of the correlate. In addition, the linear regression model was adjusted by the following covariables; sex, age, marital status, food security, wealth index, ethnicity, education of the head, area and region. The complex sampling survey design was taken into account in all multivariate regression models.

^c^The rural category included suburban population centers close to small cities, towns in rural areas distant from small towns, and disperses or very distant from rural towns

Adherence to the SP pattern in indigenous children and adults is greater than in the rest of the population.

#### Food determinants in the food transition

Additional file 1: Table S1 shows the weight of each food in the SP based on the factorial loads. Table 3 shows the differences in the five-year period in the frequency/day of food consumption. In the SP, the most influential foods that decreased in consumption in children were candy, sweets, and packaged foods. The most influential foods that decreased in consumption in adults were candy, sweets, soda, SSB, and packaged foods.

**Table 3:** Crude and adjusted differences in the frequency of consumption (times / day) in the items with the highest factorial load (L) in the period 2010–2015

Snack Pattern	5 to 17y		18 to 64y	
	Difference 1	Difference 2	Difference 1	Difference 2
Package foods	−0.09 (− 0.13, − 0,05)	−0.12 (− 0.16, − 0.08)	−0.08 (− 0.12, − 0.05)	−0.09 (− 0.12, − 0.05)
Candy or sweets	−0.09 (− 0.15, − 0.02)	−0.14 (− 0.21, − 0.08)	−0.16 (− 0.22, − 0.11)	−0.20 (− 0.25, − 0.14)
Soft drinks	0.00 (− 0.05, 0.05)	−0.01 (− 0.06 0.05)	−0.03 (− 0.09, 0.02)	−0.05 (− 0.10, 0.00)
Sausages	0.02 (− 0,01, 0.05)	0.01 (− 0.02, 0.04)	0.02 (− 0.02, 0.06)	0.02 (− 0.01, 0.05)
Fast food	0.00 (− 0.01, 0.02)	0.00 (− 0.01, 0.02)	−0.02 (− 0.04, − 0.00)	−0.02 (− 0.04, 0.00)

All calculations incorporated the complex design of the sample. L Based on analysis of factors with the frequency of consumption - times / day. Difference1. Crude difference between the frequency of day and year: 2015–2010. Difference2. Difference adjusted for sociodemographic variables between frequency / day and year: 2015–2010; Covariables used for adjustment in children, sex, age, nutrition status (based on BMI), food security, region, ethnicity, area and wealth index. Covariables used for adjustment in adults, sex, age, marital status, food security, region, schooling of the head of household, ethnicity, area and wealth index

## Discussion

In the quinquennium studied consumption in the SP decreased. The decrease was directly associated with the wealth index and education level of the head of the household. The decrease in SP consumption reflects that an adjustment in the food consumed, in favor of a better diet, is being made by the richest and most educated subjects in society. The greater adherence in the indigenous people is consistent with the above, this population is the poorest, the one with less education and in general marginalized by the state and society.

The decrease in the SP was consistent in all the variables studied and geographical regions. Because of a set of actions on the environment and individuals that have been systematically performed during the last 30 years in the United States, the period of 2003–2010 showed a substantial decrease in the consumption of calories derived from sugar-sweetened beverages (SSB) and snacks [35]. However, according to the World Health Organization (WHO) for the period of 2000–2013 in Colombia, sales of ultra-processed foods had a sustained growth of 1.9% [36]. This increasing trend was shown in 12 countries in the Americas region, except in Argentina, where the decrease in snack consumption is explained by the economic crisis suffered by that country. In this same period (2000–2013), snack sales grew by 6.1% in Asia and the Asian Pacific, 3.1% in Latin America, and 0.2% in the US. According to the WHO, Colombia comprises 16.8% of the global snack market [36]. The previous figures for Colombia do not include the possible effect of the messages and media campaigns against the consumption of snacks, sugar, soft drinks, and SSB that increased and remained visible to the entire population during 2014–2017. The above, although in Colombia there is no related public policy and a failed attempt by the Ministry of Health to tax the consumption of soft drinks and SSB [37]. The results presented here are not contradictory with those reported by the WHO because difficulty in comparison was previously noted when defining what a snack is and how to measure their consumption [25].

In both ENSINs, the decrease in snack consumption was greater in men. The consumption of snacks does not differ by sex, but in adults, snack consumption depends on the emotional state; for example, it increases in women with symptoms of depression, whereas it increases in men when they do not have symptoms of depression [38]. The above discussion is beyond the scope of this study. The greatest decrease was observed in the 18- to 24-year-old age group, which may have occurred because adolescents have the highest consumption of soda and snacks in general [39, 40]. In addition, 18 to 24 years is the age group where messages and media actions have traditionally focused [41, 42]. Clearly, as food habits are established with age, the decrease in the SP consumption is lower.

The finding that the SP consumption decreased more in subjects with a better BMI, households with food security, and the most educated and wealthiest illustrate several novel phenomena: a) inequality exists when incorporating better decisions regarding the purchase and consumption of foods that are negatively related to the state of health; b) subjects with greater “capacities/resources” translate information into better decisions [43, 44]; c) it is possible that the consumption of SP in the richest and most educated is not constitutive of the basic diet and therefore expendable, while, in the poorest, this consumption is a constitutive part of the basic diet and, therefore, less expendable; d) the wealthy abandon snacks and likely increase their fruit-vegetable/fiber consumption; e) it is possible that in the poorest, the cost of the basic food basket increases significantly, which causes them to substitute snack foods for other foods more dense in nutrients, such as fruits, vegetables or milk, but which are also more expensive [45]; f) Undoubtedly, media campaigns have a positive effect, they focused on the harmful effects of free sugar on beverages and the energy density of packaged foods. In these two items, there was the greatest decrease in consumption (Table 3); and finally g) the geographic region behaves as a proximal variable or cluster of economic and structural development and illustrates the same findings at the ecological level and the individual level: there is a gradient in the decrease in the SP consumption as the level of human and economic development in the regions increases.

Despite the effort made by the transnationals and snack producing and distributing companies to introduce snack consumption in rural areas [46], consumption still predominantly occurs in urban areas, which is why the greatest decrease was observed in those areas. However, although consumption is lower in the indigenous population, it is increasing (Table 1). The indigenous population inhabits predominantly in rural and dispersed areas.

The decrease in SP can be explained in part by a local and mediatic phenomenon derived from the attempt to reduce soda consumption [37] and, in general, by the incorporation of messages against the consumption of sugar, packaged foods, sweets, and candy in favor of a healthy diet, which do not escape globalization. Currently, due to the media, it is impossible to think that we do not have permanent campaigns against and in favor of a healthy diet [47]. The decrease in the consumption of some of the items that comprise the traditional pattern/starch, *panela* [unrefined whole cane sugar], sugar, honey, and tubers or bananas also suggests that the subjects associate snack and starch consumption with overweight and obesity, which is an association that, among others, was the axis of the aforementioned media campaigns.

The implications of total energy intake (TEI) are crucial in analyses of nutrients or food groups as exposures due to extraneous variation; we do not feel it is imperative in analyses of dietary patterns. When dietary patterns are the exposure of interest, TEI adjustment is not warranted as it could be an intermediate variable between adherence to the patterns and the outcomes. When the patterns are the outcome as in this case, extraneous variation by TEI should not affect the estimates of mean change in adherence, although precision may be lower, which in our case would represent a conservative confidence estimate.

### Implications of change in the SP

The evidence of a food transition that coexists with the nutritional transition allows for a better understanding of the latter [48]. The implications of changes in SP adherence are difficult to anticipate, and their effects require time to be observed. In Chile, for example, the study of food change raises the need to rethink the relationship between industry, government, and the health of the population [49]. In Mexico, the food transition they experience is hypothesized to result in changes in the incidence of mediators of chronic disease such as hypertension [6]. For the first time, the US has substantially decreased the consumption of calories derived from SSB and snacks [35]. In Colombia, the bidirectional relationship between BMI and dietary consumption leads us to believe that the overweight pandemic will not reach the figures or stages known in more developed societies [14]. The food transition established in the SP will allow the establishment of new strategies to control the excess of weight in the subjects and enriches the context of the nutritional transition experienced in the twenty-first century by developing countries such as Colombia.

#### Scope and limitations of the study

The FFQ is the most used methodology in nutritional epidemiology to estimate the usual consumption. A short food checklist is preferable to a long one because it avoids the fatigue of the respondent. Also, when a subject has established consumption habits the FFQ is accurate and valid [2]. The training of the pollsters in the ENSIN was directed to reduce or avoid the possible memory bias.

The results of this study are unable to establish causal relationships. Given the independence in food patterns and that the results are consistent with other food transitions and coherent with the phenomenon of the nutritional transition that was recently explained for this same population [48], where the richest migrate toward consumptions valued as healthy, such as fruits and vegetables. For the above, the occurrence of systematic information bias is unlikely. The main strength of this study is that it used data on dietary consumption obtained in two representative national surveys.

## Conclusions

In summary, there are three food consumption patterns in Colombia, and we demonstrate the transition that occurs in the SP. Both at an individual and ecological level, the region, the wealthiest, the best BMI and the most educated displayed the greatest decrease in the SP. It is plausible that both local and global media messages and campaigns have influenced this decrease in SP consumption in Colombia. Identifying the causes and the context in which this decrease occurred can help develop adequate policies and interventions to sustain this decline.

## Supplementary information

**Additional file 1 **: **Table S1.** Loading factors (L) of foods in each pattern Colombia, 2010–2015. Table S1 presents the factorial loads of the patterns established in each year. This information is of interest to the reader because it allows ensuring the comparability of the results and also guarantees the grouping in the period studied.

## Data Availability

The databases that allowed this analysis are available for public access and can be obtained by requesting them from the Ministry of Public Health of Colombia. The data generated and analyzed for the current study are available at reasonable request to the corresponding author.

## Abbreviations

BMI: Body Mass Index
ELCSA: Latin American and Caribbean Scale of Food Security
ENSIN: National Surveys of the Nutritional Situation
FFQ: Food Frequency Questionnaire
ICBF: Colombian Family Welfare Institute
SCF: Subcutaneous Fat
SP: Snack Pattern
SSB: Sugar Sweetened Beverages
TEI: Total Energy Intake
WHO: World Health Organization

## References

[1] Hu FB (2002). Dietary pattern analysis: a new direction in nutritional epidemiology. Curr Opin Lipidol.

[2] Willett W (2013). Nutritional epidemiology.

[3] Lozada AL, Flores M, Rodríguez S (2007). Dietary patterns in Mexican adolescent girls. A comparison of two methods. National Nutrition Survey, 1999. Salud Publica Mex.

[4] Herrán OF, Patiño GA, Del Castillo SE (2016). Dietary transition and excess weight in adults according to the Encuesta de la Situación Nutricional en Colombia, 2010. Biomédica..

[5] Moreno-Altamirano L, Hernández-Montoya D, Silberman M (2014). The nutrition transition and the double burden of malnutrition: changes in dietary patterns 1961-2009 in the Mexican socioeconomic context. Arch Latinoam Nutr.

[6] Monge A, Lajous M, Ortiz-Panozo E (2018). Western and modern Mexican dietary patterns are directly associated with incident hypertension in Mexican women: a prospective follow-up study. Nutr J.

[7] Nasreddine L, Tamim H, Itani L (2018). A minimally processed dietary pattern is associated with lower odds of metabolic syndrome among Lebanese adults. Public Health Nutr.

[8] Grasgruber P, Sebera M, Hrazdira E (2016). Food consumption and the actual statistics of cardiovascular diseases: an epidemiological comparison of 42 European countries. Food Nutr Res.

[9] Wang D, Hawley NL, Thompson AA (2017). Dietary patterns are associated with metabolic outcomes among adult Samoans in a cross-sectional study. J Nutr.

[10] Davis C, Bryan J, Hodgson J (2015). Definition of the Mediterranean diet; a literature review. Nutrients..

[11] León-Muñoz LM, Guallar-Castillón P, Graciani A (2012). Adherence to the Mediterranean diet pattern has declined in Spanish adults. J Nutr.

[12] Deutch B, Dyerberg J, Pedersen HS (2007). Traditional and modern Greenlandic food — dietary composition, nutrients and contaminants. Sci Total Environ.

[13] Albuquerque RC, Baltar VT, Marchioni DM (2014). Breast cancer and dietary patterns: a systematic review. Nutr Rev.

[14] Popkin BM, Adair LS, Ng SW (2012). Global nutrition transition and the pandemic of obesity in developing countries. Nutr Rev.

[15] Manzel A, Muller DN, Hafler DA (2014). Role of “Western diet” in inflammatory autoimmune diseases. Curr Allergy Asthma Rep.

[16] Delisle H (2010). Findings on dietary patterns in different groups of African origin undergoing nutrition transition. Appl Physiol Nutr Metab.

[17] Myhre JB, Løken EB, Wandel M (2015). The contribution of snacks to dietary intake and their association with eating location among Norwegian adults – results from a cross-sectional dietary survey. BMC Public Health.

[18] van Ansem WJC, van Lenthe FJ, Schrijvers CTM (2014). Socio-economic inequalities in children’s snack consumption and sugar-sweetened beverage consumption: the contribution of home environmental factors. Br J Nutr.

[19] Hess J, Slavin J (2014). Snacking for a cause: nutritional insufficiencies and excesses of U.S. Children, a critical review of food consumption patterns and macronutrient and micronutrient intake of U.S. children. Nutrients.

[20] McDonald CM, Baylin A, Arsenault JE (2009). Overweight is more prevalent than stunting and is associated with socioeconomic status, maternal obesity, and a snacking dietary pattern in school children from Bogota, Colombia. J Nutr.

[21] Shroff MR, Perng W, Baylin A (2014). Adherence to a snacking dietary pattern and soda intake are related to the development of adiposity: a prospective study in school-age children. Public Health Nutr.

[22] Ambrosini GL, Emmett PM, Northstone K (2012). Identification of a dietary pattern prospectively associated with increased adiposity during childhood and adolescence. Int J Obes.

[23] O’Connor L, Brage S, Griffin SJ (2015). The cross-sectional association between snacking behaviour and measures of adiposity: the fenland study, UK. Br J Nutr.

[24] Cordain L, Eaton SB, Sebastian A (2005). Origins and evolution of the Western diet: health implications for the 21st century. Am J Clin Nutr.

[25] Hess JM, Jonnalagadda SS, Slavin JL (2016). What is a snack, why do we snack, and how can we choose better snacks? A review of the definitions of snacking, motivations to snack, contributions to dietary intake, and recommendations for improvement. Adv Nutr.

[26] Ocampo PR, Prada GE, Herrán OF (2015). Patrones de consumo alimentario y exceso de peso infantil; encuesta de la situación nutricional en Colombia, 2010. Rev Chil Nutr.

[27] Instituto Nacional de Salud, Universidad de Antioquia, OPS, Profamilia, Instituto Colombiano de Bienestar Familiar (2006). Encuesta Nacional de la Situación Nutricional de Colombia.

[28] Instituto Colombiano de Bienestar Familiar (2010). Resumen Ejecutivo Encuesta Nacional de la Situación Nutricional en Colombia, ENSIN 2010.

[29] Bautista LE, Herrán OF, Pryer JA (2005). Development and simulated validation of a food-frequency questionnaire for the Colombian population. Public Health Nutr.

[30] Herrán OF, Ardila MF (2006). Validity and reproducibility of two semi-quantitative alcohol frequency questionnaires for the Colombian population. Public Health Nutr.

[31] WHO (2007). Growth reference 5–19 years.

[32] FAO (2012). Escala Latinoamericana y Caribeña de Seguridad Alimentaria (ELCSA) - Manual de uso y aplicación.

[33] Rutstein SO. The DHS wealth index: approaches for rural and urban areas. Demogr Health Res. 2008;60:1–22. https://dhsprogram.com/pubs/pdf/WP60/WP60.pdf. Accessed 18 July 2018.

[34] StataCorp S (2015). Statistical software: release 14.

[35] Bleich SN, Wolfson JA (2015). Trends in SSBs and snack consumption among children by age, body weight, and race/ethnicity. Obesity..

[36] OPS, OMS (2015). Alimentos y bebidas ultraprocesados en América Latina: tendencias, efecto sobre la obesidad e implicaciones para las políticas públicas.

[37] Ortiz-Castaño D (2016). Impuesto a gaseosas en Colombia: Postobón responde - el Colombiano [Internet].

[38] Camilleri GM, Méjean C, Kesse-Guyot E (2014). The associations between emotional eating and consumption of energy-dense snack foods are modified by sex and depressive symptomatology. J Nutr.

[39] Villamor E, Quintero-Lesmes DC, Herran OF (2017). Intake of soft drinks and sugar sweetened beverages by Colombian children and adolescents. Rev Bras Saude Mater Infant.

[40] Malik VS, Schulze MB, Hu FB (2006). Intake of sugar-sweetened beverages and weight gain: a systematic review. Am J Clin Nutr.

[41] Beets MW, Tilley F, Kim Y (2011). Nutritional policies and standards for snacks served in after-school programmes: a review. Public Health Nutr.

[42] Beets MW, Glenn Weaver R, Turner-McGrievy G (2014). Making healthy eating and physical activity policy practice: the design and overview of a group randomized controlled trial in afterschool programs. Contemp Clin Trials.

[43] Sen A (2002). ¿Por qué la equidad en salud?. Rev Panamericana Salud Pública.

[44] Sen A, Rabasco E, Toharia L (2000). Desarrollo y libertad.

[45] Cameron AJ, Thornton LE, McNaughton SA (2013). Variation in supermarket exposure to energy-dense snack foods by socio-economic position. Public Health Nutr.

[46] Mahajan V, Warbelow K (2016). Rise of rural consumers in developing countries : harvesting 3 billion aspirations.

[47] Harris JL, Bargh JA (2009). Television viewing and unhealthy diet: implications for children and media interventions. Health Commun.

[48] Kasper NM, Villamor E, Herrán OF (2009). Obesity prevalence in Colombian adults is increasing fastest in lower socio-economic status groups and urban residents: results from two nationally representative surveys. Public Health Nutr.

[49] Crovetto M, Uauy R (2012). Changes in processed food expenditure in the population of metropolitan Santiago in the last twenty years. Rev Med Chil.

