# Supplementary material for: Adherence to a snacking dietary pattern is decreasing in Colombia among the youngest and the wealthiest: results of two representative national surveys
Source: BMC Public Health. 2019 Dec 19;19:1702. doi: 10.1186/s12889-019-8057-6 (PMC6921475; doi:10.1186/s12889-019-8057-6)
Supplement: Supplementary file 1 — Additional file 1 : Table S1. Loading factors (L) of foods in each pattern Colombia, 2010–2015. Table S1 presents the factorial loads of the patterns established in each year. This information is of interest to the reader because it allows ensuring the comparability of the results and also guarantees the grouping in the period studied. [file 12889_2019_8057_MOESM1_ESM.docx]

**Table S1.** Loading factors (L) of foods in each pattern Colombia, 2010-2015.

| Pattern / Ítems | L | | |
| --- | --- | --- | --- |
|  | 2010 | 2015 |  |
| **Fruit-vegetable/dairy** |  |  |  |
| Milk (liquid or powder) | 0.41 | 0.39 |  |
| Cheese, kumis, yogurt, cream cheese ... | 0.41 | 0.43 |  |
| Raw vegetables | 0.46 | 0.42 |  |
| Cooked vegetables | 0.41 | 0.40 |  |
| Whole fruits | 0.43 | 0.46 |  |
| Fruits in juice | 0.53 | 0.48 |  |
| Bread, Arepa or Cookies | 0.25 | 0.31 |  |
| Whole-grain foods | 0.35 | 0.31 |  |
| Chicken | 0.25 | 0.37 |  |
| Black pudding or beef viscera | 0.17 | 0.21 |  |
| Low calorie foods (light) | 0.25 | 0.19 |  |
| Tuna or sardines | 0.13 | 0.22 |  |
| **Snack** |  |  |  |
| Package foods | 0.46 | 0.56 |  |
| Sweets or sweets | 0.40 | 0.56 |  |
| Soft drinks or soft drinks (powder, box, bottle) | 0.47 | 0.50 |  |
| Fast food | 0.42 | 0.40 |  |
| Butter, cream, butter | 0.11 | 0.16 |  |
| Sausages | 0.44 | 0.42 |  |
| Chicken giblets | 0.08 | 0.08 |  |
| **Traditional / starch** |  |  |  |
| Panela, sugar, honey | 047 | 0.49 |  |
| Rice or pasta | 0.46 | 0.48 |  |
| Fried foods | 0.43 | 0.47 |  |
| Dry beans | 0.33 | 0.37 |  |
| Tubers or banana | 0.27 | 0.40 |  |
| Eggs | 0.33 | 0.33 |  |
| Beef, veal, pork ... | 0.31 | 0.38 |  |
| Fish or seafood | -0.17 | -0.04 |  |
| Coffee or tea | 0.21 | 0.24 |  |
| **L** Based on analysis of factors with the frequency of consumption times / day. | | |  |
